# Mathematical Modeling of Bacterial Kinetics to Predict the Impact of Antibiotic Colonic Exposure and Treatment Duration on the Amount of Resistant Enterobacteria Excreted

**DOI:** 10.1371/journal.pcbi.1003840

**Published:** 2014-09-11

**Authors:** Thu Thuy Nguyen, Jeremie Guedj, Elisabeth Chachaty, Jean de Gunzburg, Antoine Andremont, France Mentré

**Affiliations:** 1 IAME, UMR 1137, INSERM, Paris, France; 2 IAME, UMR 1137, Univ Paris Diderot, Sorbonne Paris Cité, Paris, France; 3 Institut Gustave-Roussy, Villejuif, France; 4 Da Volterra, Paris, France; 5 AP-HP, Hôpital Bichat, Paris, France; University of New South Wales, Australia

## Abstract

Fecal excretion of antibiotics and resistant bacteria in the environment are major public health threats associated with extensive farming and modern medical care. Innovative strategies that can reduce the intestinal antibiotic concentrations during treatments are in development. However, the effect of lower exposure on the amount of resistant enterobacteria excreted has not been quantified, making it difficult to anticipate the impact of these strategies. Here, we introduce a bacterial kinetic model to capture the complex relationships between drug exposure, loss of susceptible enterobacteria and growth of resistant strains in the feces of piglets receiving placebo, 1.5 or 15 mg/kg/day ciprofloxacin, a fluoroquinolone, for 5 days. The model could well describe the kinetics of drug susceptible and resistant enterobacteria observed during treatment, and up to 22 days after treatment cessation. Next, the model was used to predict the expected amount of resistant enterobacteria excreted over an average piglet's lifetime (150 days) when varying drug exposure and treatment duration. For the clinically relevant dose of 15 mg/kg/day for 5 days, the total amount of resistant enterobacteria excreted was predicted to be reduced by 75% and 98% when reducing treatment duration to 3 and 1 day treatment, respectively. Alternatively, for a fixed 5-days treatment, the level of resistance excreted could be reduced by 18%, 33%, 57.5% and 97% if 3, 5, 10 and 30 times lower levels of colonic drug concentrations were achieved, respectively. This characterization on *in vivo* data of the dynamics of resistance to antibiotics in the colonic flora could provide new insights into the mechanism of dissemination of resistance and can be used to design strategies aiming to reduce it.

## Author Summary

Fecal excretion of antibiotics and resistant bacteria in the environment are major public health threats associated with extensive farming. Innovative strategies that reduce the intestinal antibiotic concentrations during treatment are in development and could help prevent the dissemination of resistance. In order to anticipate the impact of these strategies, the effect of lower exposure on the amount of resistant enterobacteria excreted needs to be quantified precisely. Here, we introduce a bacterial kinetic model to capture the complex relationships between dosage regimen, antibiotic fecal concentrations, loss of susceptible enterobacteria and growth of resistant strains in the feces of piglets receiving different doses of ciprofloxacin for 5 days. We use this model to evaluate by simulation how much it would be necessary to reduce the antibiotic colonic concentration in order to prevent the expansion of antibiotic resistance. This approach provides new insights into the mechanism of dissemination of resistance during treatments and can be used to design strategies to reduce it.

## Introduction

Antibiotics are widely used in animal farming for curative, prophylaxis and metaphylaxis purposes. This results in massive excretion of antibiotics [1], [2] and resistant bacteria with the feces of the animals during treatments [3]. It impacts the ecology of the environment and ultimately contributes to increase resistance in bacteria infecting humans [4], making resistance of human bacteria to antibiotics one of the major threats to public health in the next decade [5], [6].

In particular, fluroquinolones (FQ) are widely used in animals, including in pets and farm animals for respiratory, urinary tract and skin infections, and have also been categorized as critical for human use (see the WHO list of Critically Important Antimicrobials [7]). Unfortunately resistance to FQ has regularly increased over the last decades and has reached a level that jeopardizes the treatment of common human infections caused by members of the *Enterobacteriaceae* family (enterobacteria), in particular *Escherichia coli* and *Klebsiella spp*, such as gastrointestinal and urinary tract infections [8], [9]. Besides causing infections, enterobacteria are also naturally present in the intestinal commensal flora of humans and several animal species [10], [11]. When a subject is treated with FQ, either by the oral or the parenteral route, a fraction of the dose administered is eliminated in the intestine after biliary and intestinal excretion [12]. These residual concentrations may be sufficient to eliminate FQ-susceptible species but not to act against resistant enterobacteria [13], [14]. Consequently, resistant enterobacteria can multiply in these free niches and reach high concentrations before being excreted in the feces [15]. This set of events is believed to be a major driver of emergence and dissemination of bacterial resistance [16] and this is why innovative strategies, such as charcoal-based adsorbent, are now being developed to reduce intestinal antibiotic residues [17], [18]. However, the effect of lower antibiotic exposure on the amount of resistant enterobacteria excreted has not been characterized, making it difficult to anticipate the impact of these strategies.

We previously reported that intestinal excretion of ciprofloxacin (a FQ) resistant enterobacteria increased with the colonic exposure to ciprofloxacin in piglets [15]. Here, using a mechanistic model, we now aim to characterize the complex relationships between antibiotic dosage regimen, pharmacokinetics in feces, loss of susceptible enterobacteria and growth of resistant strains. This approach has mostly been used to fit *in vitro* data during antibiotic treatment [19]–[24]. To the best of our knowledge, only very few papers aimed to fit *in vivo* bacterial kinetic data (see [25] for instance) and it has never been used to characterize the dynamic of enterobacteria in the intestinal flora during treatment. This lack of data may be due to the difficulty to obtain and analyze such data, characterized by a high level of variability both in drug concentrations and in bacterial counts [15].

This difficulty can be in part circumvented by using nonlinear mixed-effect models (NLMEM), a statistical approach that optimally uses all the information available in a population sample, including the between subject variability, in order to increase the ability to estimate model parameters [26]. Here, we used this technique to fit a dynamic mathematical model to the kinetics of drug concentrations and the counts of total and resistant fecal enterobacteria. After the model parameters have been estimated, a large number of scenarios can be evaluated *in silico* and this model was then used to predict the effect of reduced intestinal antibiotic concentrations on the amounts of FQ resistant enterobacteria excreted.

## Materials and Methods

### Ethics statement

The protocol was approved by Pharnimal (Eghezée, Belgium) and Animal Breeding Parteners facilities - Faculty of Veterinary Medicine (Liège, Belgium). Animal housing and care comply with the guidelines of the local ethical committee, in accordance with EU Guideline on Good Clinical Practice for the conduct of Clinical trials and Veterinary Medicinal Products (Eudralex. Volume 7A: 7AE1a) and VICH guideline on Good Clinical Practice (VICH GL9).

### Experimental data

The data we used are from a prospective randomized study previously published [15]. Briefly, 29 piglets from a single farm were included 4 weeks after birth and were put in individual cages throughout the study and randomly assigned (9∶10∶10) to once-a-day oral treatment with placebo, ciprofloxacin 1.5 or 15 mg/kg/day for 5 days (D1 to D5).

Ciprofloxacin concentrations and counts of total and ciprofloxacin resistant enterobacteria were measured in fecal samples at pre-dose on D1, D3 and D5 and on D7, D9, D12, D16 and D27. A microbiological assay that measures the ability of the antibiotic to inhibit the growth of *Bacillus subtilis* strain ATCC6633 was used to determine fecal concentrations of free (active) ciprofloxacin. Total and fluoroquinolones resistant enterobacteria were counted by plating serial dilutions of the feces on Drigalski agar without or with 2 mg/L of ciprofloxacin, respectively [15]. The 2 mg/L concentration was chosen in agreement with the EUCAST clinical breakpoints (www.eucast.org). The limit of detection was 0.1 µg/g and 10^2^ CFU/g of feces for antibiotic concentrations and bacterial counts, respectively. The data will be available upon request.

### Mathematical model of bacterial kinetics

#### Pharmacokinetics (PK) of ciprofloxacin residues

Fecal ciprofloxacin concentrations were fitted using a one compartment model with first order elimination of rate *k_e_*. Although the drug is given orally once a day, we assumed that ciprofloxacin arrived in the colon and the feces with a constant rate (*qD/V*) from the start of therapy (D1) until one day after its end (D6). The equation of the model is given by:
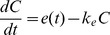
(1)where *e(t) = qD/V* if *t*<6 days and *e(t)* = 0 otherwise, with *D* being the daily dose of ciprofloxacin administered, *q* the fraction of the dose which reached the intestinal tract, and *V* the volume of distribution. Only the ratio *V/q* can be estimated from the data and we assume in the following *q* = 1 without loss of generality. Consequently, the fecal concentrations reach a plateau whose level is proportional to the dose, given by *C*_ss_ = *D/(k_e_V)*.

#### Origin of resistant enterobacteria

The presence of resistant bacteria can result from *de novo* spontaneous mutations [27] or from the ingestion of susceptible and ciprofloxacin-resistant bacteria [28]. However, the mutation frequency in enterobacteria is about 10^−8^ to 10^−6^ per cycle and at least two rounds of mutations are needed to confer resistance to ciprofloxacin above 2 mg/L in enterobacteria [29]–[31]. Thus, assuming that the total number of enterobacteria in the digestive tract is about 10^6^–10^8^ CFU/g [32], the probability of occurrence of spontaneous mutations that confer resistance are extremely small [33]. Yet almost all piglets (25/29) had large number of resistant enterobacteria at baseline >10^2^ CFU/g. This high prevalence of resistance at baseline (i.e., in absence of any therapeutic pressure, advantage of selection or history of treatment) is therefore unlikely to be explained by the rate of mutations. This is why we assumed in our main analysis that resistant bacteria were due to exogenous factors (e.g., ingestion) rather than mutations. Models including mutation as the cause of resistance are presented in the supporting information.

#### Kinetic modeling of susceptible and resistant enterobacteria in feces

**Figure 1:**
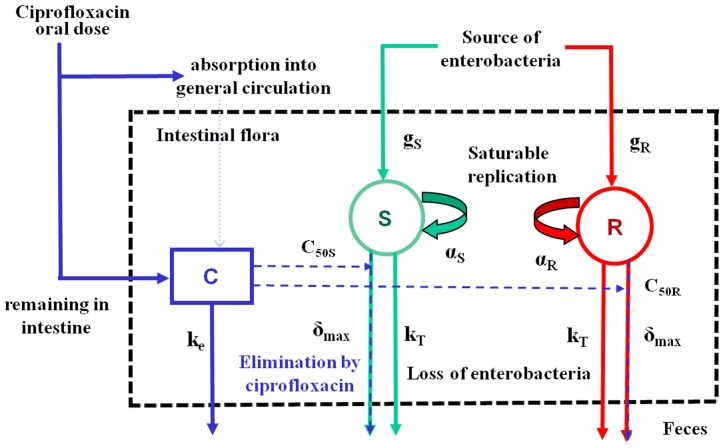
Model used for the kinetics of susceptible (S) and resistant (R) enterobacteria in presence of ciprofloxacin (C) in intestinal flora. *k_e_* is the elimination rate constant of intestinal ciprofloxacin concentrations; *g*_S_ and *g*_R_ are constant sources of susceptible and resistant bacteria respectively coming from outside; *k*_T_ is the rate constant of enterobacteria loss in absence of treatment; δ_max_ is the maximal killing rate of enterobacteria by ciprofloxacin; *C*_50S_ and *C*_50R_ are the ciprofloxacin concentrations at which 50% of the maximal killing effect occurs in susceptible and resistant enterobacteria respectively.

The evolution of the counts per gram of feces of ciprofloxacin resistant enterobacteria, R(t), and drug susceptible enterobacteria, S(t), was modeled as (Figure 1):
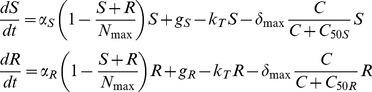
(2)where we assumed a logistic growth for S and R with constant proliferation rates *α*_S_ and *α_R_*, respectively, and a saturation term *N*_max_ representing the total maximal number of enterobacteria. By definition, the fitness of S is greater than that of R (*α*_S_≫*α_R_*) and thus the susceptible enterobacteria are in large excess at treatment initiation (S_0_≫R_0_). Moreover the arrival of S and R in the digestive tract was assumed to occur using a zero order process with values *g*_S_ and *g_R_* respectively. In absence of treatment, both populations were supposed to be in equilibrium and equal to S_0_ and R_0_, respectively, given by:
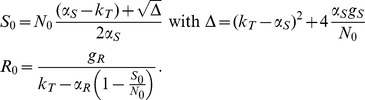
It can easily be shown that 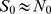 and 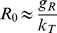 (see Supporting Information for more details).

When ciprofloxacin residues reach the colon, susceptible and resistant enterobacteria are eliminated in a ciprofloxacin concentration dependent rate, described by an E-max function given by 
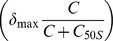
 and 
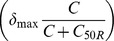
, respectively, where *C*_50S_ and *C*_50R_ are the ciprofloxacin concentrations corresponding to 50% of the maximal eliminating effect, noted *δ*_max_.

**Figure 2:**
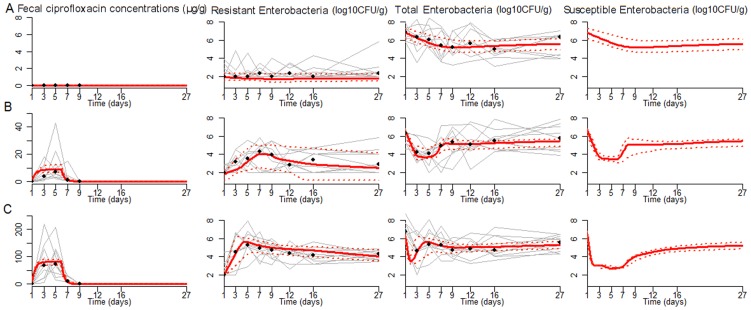
Experimental data from individual piglets (grey lines) and observed medians (black dots) versus medians predicted by the model (red lines) for fecal ciprofloxacin concentrations (first column), resistant (second column), total (third column) and susceptible enterobacteria (fourth column) in three treatment groups: A) placebo, B) ciprofloxacin 1.5 mg/kg/day, C) ciprofloxacin 15 mg/kg/day. The red dotted lines represent the 10% and 90% quantiles of the estimated individual curves.

Moreover, because the amount of total enterobacteria, S+R, was not constant in the placebo group (no treatment) and showed a decrease followed by an increase (Figure 2), *N*_max_ was modeled using a biphasic function:
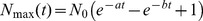
(3)where *N*_0_ was the total maximal enterobacterial population at baseline, *a* and *b* were the parameters characterizing the decreasing and increasing slopes of *N*_max_ changes.

### Parameter estimation

Because the sensitivity of resistant enterobacteria to treatment could not be precisely identified (not shown), we estimated only C_50S_ and we fixed the ratio C_50R_/C_50S_ to various putative values equal to 4, 16, 100 and ∞ (i.e., ciprofloxacin had no activity in resistant enterobacteria and *δ*_max_*C/(C+C_50R_) was fixed to 0).

For each case, ciprofloxacin concentrations and counts of resistant and total enterobacteria observed in the experimental animals were simultaneously fitted using nonlinear mixed effect models (NLMEM), an approach which borrows strength from the whole sample to precisely estimate the population parameters, such as the mean and the between-subject variability (BSV) [34]. In this approach, each individual parameter *θ*_i_ of piglet *i* is modeled as a fixed part *θ*, which represents the median value of the parameter in the population, and a random part *η*_i_ chosen from a Gaussian distribution with mean 0 and standard deviation *ω* that accounts for the BSV. Therefore, all parameters can be written as 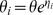 where 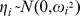. Parameter variability was fixed to 0 in case of low estimated value and high relative standard error (RSE). We assumed combined error model for fecal ciprofloxacin concentrations (parameterized in 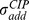 and 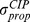 for additive and proportional error, respectively), and constant error model for log_10_ of resistant and total counts of enterobacteria, noted 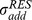 and 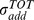, respectively.

Data were analyzed using MONOLIX 4.2.0 (www.lixoft.eu), a software devoted to maximum likelihood estimation of parameters in NLMEM, based on the SAEM algorithm [35]. The code for the model implemented in MONOLIX can be found in the supporting information. Details on the fitting method and the likelihood expression in kinetic models defined by ordinary differential equations can be found in [36]. Of note, maximum likelihood estimation can take into account the information brought by data under the level of detection [37]. After the population parameters were determined, the values of the parameters for individual piglets were deduced using empirical Bayes estimates, and predicted medians at each timepoint could be obtained for the resistant and total enterobacteria as well as for the susceptible ones. Model evaluation was done by analyzing the distribution of the residuals and by comparing the observed and predicted median values. The data fitting obtained for the different putative values of the ratio C_50R_/C_50S_ were compared using the Bayesian Information Criterion (BIC, the lower the better), a fitting criterion that accounts for the number of parameters.

### Simulation and prediction

Using the estimated distributions of the parameters with the different putative values of the ratio C_50R_/C_50S_, we performed a Monte Carlo simulation to predict the amounts of resistant enterobacteria that would be excreted with similar or lower fecal concentrations of ciprofloxacin than those observed in the experimental piglets and with various treatment duration of 1, 3, 5 and 10 days. For each scenario, parameters for 1000 piglets were generated. Because the colonic drug concentration rapidly reaches a dose-proportional plateau level, C_ss_, the effects of a x-fold lower drug exposure were obtained by simulating the model with a x-fold lower administered dose. For instance, the effect of a strategy that could reduce colonic drug concentrations by 99% as compared to the therapeutic dose of 15 mg/kg/day were obtained by simulating the model with a dose of 0.15 mg/kg/day.

Of note, in order to facilitate comparison between these scenarios, we assumed constant value for the maximal bacteria density *N*_max_ (i.e., *a = b* = 0 in equation 3) in the simulation study. We calculated at each timepoint the median value for the antibiotic residual concentrations, C_med_, as well as the median counts of susceptible, resistant and total enterobacteria, noted S_med_, R_med_ and T_med_, respectively.

Further, we assumed an average remaining lifespan of 150 days (5 months) after the initiation of the treatment [38] and an excretion of about 100 g of feces per day [39]. Therefore, for a given treatment duration and a given drug exposure, the median total amount of resistant bacteria excreted from the beginning of the treatment (t = 0) until death, AR, is given by 
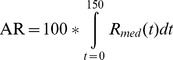
. Let AR_0_ and AR_15_ be the corresponding amounts excreted if there was no drug exposure and the reference exposure (i.e., corresponding to the therapeutic dose of 15 mg/kg/day), respectively. Then the normalized reduction of the amounts of resistant enterobacteria excreted for a given dose and treatment duration is given by 
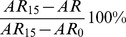
.

## Results

### Pharmacokinetic model of intestinal ciprofloxacin concentrations

The PK model described well the rapid increase in fecal concentrations of ciprofloxacin followed by a plateau (Figure 2, Figure S1 and Figure S2). The mean elimination rate constant of intestinal ciprofloxacin concentrations, *k_e_*, was equal to 1.97 day^−1^, corresponding to a half-life of about 8 hours (Table 1). The plateau exposure, *C*_ss_, was estimated at 8.7 µg/g in the animals treated with the dose 1.5 mg/kg/day and at 87 µg/g in those treated with 15 mg/kg/day.

**Table 1:** Population parameter estimates and relative standard errors (RSE, in %) of the bacterial kinetic model.

Fixed effects	Estimates	RSE(%)	Variabilities	Estimates	RSE(%)
*V* (L/kg)	0.088	11	ω_V_	0.30	32
*k_e_* (day^−1^)	1.97	8	ω_ke_	0.26	30
log_10_ *N*_0_ (logCFU/g)	6.56	2	*ω*log_10_ *N*_0_	0.08	21
a	0.56	13	ω_a_	0.52	21
b	0.02 10^−1^	38	ω_b_	0.36	277
*α*_S_ (day^−1^)	13.66	16	ω*_α_*_*R*_	0.93	14
*α*_R_ (day^−1^)	1.90	21	ω*_k__T_*	1.28	29
*g*_S_ (CFU/g/day)	14067	82	*ω_δ_*_max_	0.19	84
*g*_R_ (CFU/g/day)	10	22	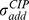 (µg/g)	0.04	26
*k*_T_ (day^−1^)	0.11	33	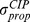	0.84	11
*δ*_max_ (day^−1^)	27.14	14	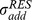 (log_10_CFU/g)	1.04	6
*C*_50S_ (µg/g)	4.91	16	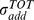 (log_10_CFU/g)	1.01	5

**NOTE.** *V* is the volume of distribution; *k_e_* is the elimination rate constant of intestinal ciprofloxacin residues; *N*_0_ is the initial maximal enterobacteria density; *a* and *b* are the two parameters characterizing the changes in the maximal enterobacteria population *N*_max_ over time; *α*_S_ and *α*_R_ are the replication rate constants, *g*_S_ and *g*_R_ are constant sources coming from outside for susceptible and resistant enterobacteria respectively; *k*_T_ is the rate constant of enterobacteria loss in absence of treatment; δ_max_ is the maximal kill rate constant; *C*_50S_ is the ciprofloxacin concentration at which 50% of the maximal killing effect occurs in susceptible enterobacteria; *ω* is the standard error of random effect; 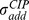 is the additive error and 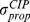 the proportional error of fecal ciprofloxacin concentrations; 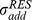 and 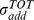 are constant errors on log_10_ of resistant and total enterobacteria counts respectively.

### Bacterial kinetic fitting

We found that the best fit was obtained when assuming that C_50R_/C_50S_ was equal to ∞, i.e., ciprofloxacin had no activity in resistant enterobacteria (Table 2). Consequently, we neglected the effect of ciprofloxacin on resistant bacteria in the final model. Interestingly almost all parameters could be estimated with a reasonable precision: lower than 30% for fixed parameters and 50% for variability terms (Table 1). g_s_ and ω_δmax_ were out of these ranges (RSE of 82% and 84%, respectively) and therefore their estimated value should be interpreted with caution.

**Table 2:** Comparison of fitting critera for different fixed values of the ratio C_50R_/C_50S_.

C_50R_/C_50S_	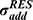	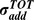	BIC
	(log_10_CFU/g)		
4	1.11	1.07	1984
16	1.08	1.06	1976
100	1.06	1.02	1976
**∞**	**1.04**	**1.01**	**1954**

**NOTE.** BIC is the Bayesian Information Criterion (the lower the better); 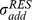 and 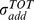 are constant errors on log_10_ of resistant and total *Enterobacteriaceae* counts respectively.

The model could well characterize the kinetics of total and resistant enterobacterial counts observed in experimental animals during and after treatment (Figure 2, Figure S1 and Figure S2). *C*_50S_ was equal to 4.91 µg/g and lower than *C*_ss_ of both dosing groups. Therefore susceptible enterobacteria decreased rapidly after initiation of treatment, with a maximal elimination rate, *δ*_max_, estimated to 27.1 day^−1^, corresponding to a half-life (ln(2)/δ_max_) of 37 minutes. In absence of treatment, both resistant and susceptible bacteria were eliminated at a much lower rate *k*_T_ equal to 0.1 day^−1^. Replication rates of resistant and susceptible enterobacteria were estimated to 1.9 day^−1^ and 13.7 day^−1^, respectively, giving a relative fitness of the resistant enterobacteria [40] of α_R_/α_S_ = 1.9/13.7 = 14% in absence of treatment.

### Prediction for various dosage regimens

**Figure 3:**
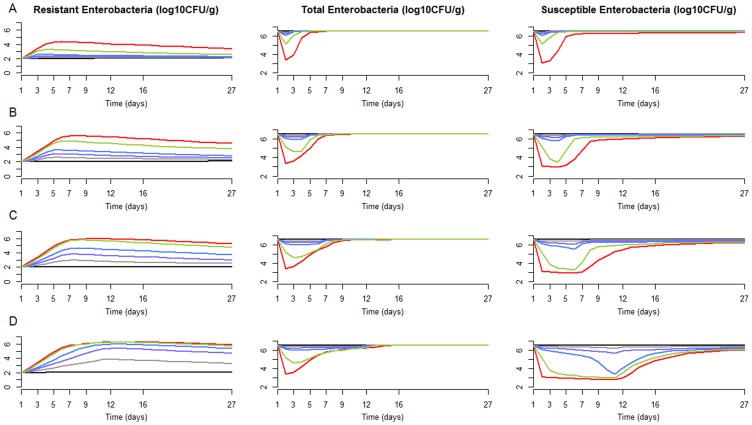
Resistant (first column), total (second column) and susceptible (third column) enterobacteria predicted for various fecal concentrations of ciprofloxacin *C_ss_*: 0 µg/g (black), 0.9 µg/g (grey), 1.8 µg/g (violet), 2.9 µg/g (blue), 8.7 µg/g (green), 87 µg/g (red), for different treatment durations: A) 1 day, B) 3 days, C) 5 days; D) 10 days.

First, we simulated the evolution of enterobacteria counts that would be obtained with similar or lower fecal concentrations of ciprofloxacin than those observed in the experimental piglets, i.e., *C_ss_* = 87, 58, 29, 18, 8.7, 2.9, 1.8, 0.9 or 0 µg/g and with treatment duration of 1, 3, 5 or 10 days (see methods). The simulated curves C_med_, R_med_, S_med_ and T_med_ are presented in Figure 3. The amount of resistant enterobacteria excreted increased with fecal concentrations of antibiotic or treatment duration. In all scenarios, including those with low exposures, susceptible strains were rapidly eliminated after treatment initiation and replaced by resistant enterobacteria within 5–10 days (Figure 3). After treatment end, resistant enterobacteria disappeared slowly and it took 2–15 days for susceptible enterobacteria to return to pre-treatment levels, consistent with observations from both dosing groups (Figure 2). As the elimination rate in absence of treatment, k_T_, was low, resistant bacteria could remain in high counts for several weeks after the end of treatment (see Figure S3).

We then used these results to calculate the total amount of resistant enterobacteria excreted over the remaining lifespan of a piglet (150 days) according to the treatment duration (Table 3). In animals exposed to the reference drug colonic concentration *C_ss_* of 87 µg/g, i.e., the one obtained with the therapeutic dose of 15 mg/kg, the predicted amounts of resistant enterobacteria excreted were equal to 7.5, 8.6 and 9.2 log_10_CFU for 1, 3, and 5 days of treatment, respectively. In other words, the level of resistance excreted can be reduced by 75% and 98% when reducing treatment durations from 5 days to 3 or 1 day, respectively.

**Table 3:** Impact of ciprofloxacin colonic exposure and treatment duration on the amount of resistant enterobacteria (R) excreted over 150 days.

Treatment duration	C_ss_ (µg/g)	% of C_ss_ reduction*	Total amount of R excreted (log_10_CFU)	% of reduction of amount of R*
1 day	87	0	7.5	0.0
	58	33	7.4	19.7
	29	67	7.2	46.8
	18	80	7.1	61.0
	8.7	90	6.6	90.4
	2.9	97	6.3	98.1
	1.8	98	6.3	99.0
	0.9	99	6.3	99.6
	0	100	6.2	100.0
3 days	87	0	8.6	0.0
	58	33	8.6	4.8
	29	67	8.4	42.3
	18	80	8.3	50.4
	8.7	90	7.8	84.4
	2.9	97	6.9	98.5
	1.8	98	6.5	99.5
	0.9	99	6.3	99.9
	0	100	6.2	100.0
5 days	87	0	9.2	0.0
	58	33	9.2	3.5
	29	67	9.1	17.7
	18	80	9.0	33.0
	8.7	90	8.8	57.5
	2.9	97	7.8	96.3
	1.8	98	7.0	99.4
	0.9	99	6.5	99.9
	0	100	6.2	100.0
10 days	87	0	9.6	0.0
	58	33	9.6	2.5
	29	67	9.5	4.6
	18	80	9.5	16.9
	8.7	90	9.5	17.0
	2.9	97	9.1	63.0
	1.8	98	8.5	91.5
	0.9	99	7.1	99.7
	0	100	6.2	100.0

C_ss_: fecal plateau concentration of ciprofloxacin.

*: compared to the value achieved with the clinically relevant dose of 15 mg/kg/day (see Methods).

**Figure 4:**
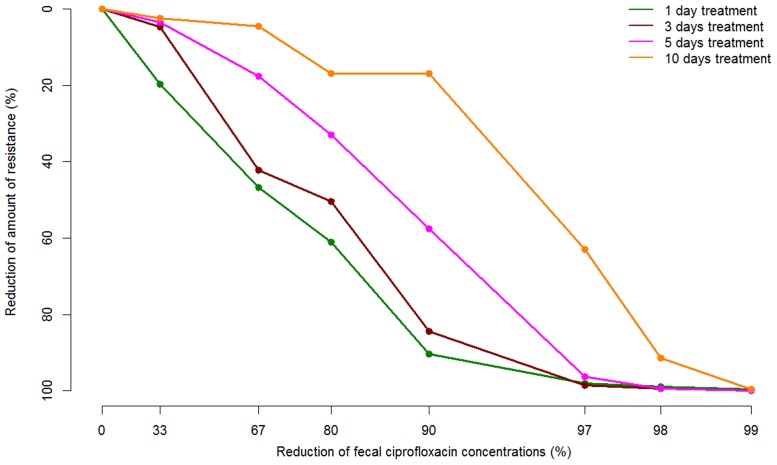
Predicted total amounts of resistant enterobacteria excreted for various levels of fecal ciprofloxacin concentrations and treatment duration. Total amounts excreted and fecal ciprofloxacin concentrations are expressed as a relative reduction from the clinically relevant dose (15 mg/kg/day).

Next, we estimated the reduction in colonic drug exposure that needs to be achieved in order to excrete 50% less resistance than with the reference drug colonic concentration of 87 µg/g. Figure 4 shows that this would require reducing drug exposure by 67%, 80% and 90% for a 1, 3 and 5 day treatment. If we focus on a 5 day treatment, reducing drug exposure by 80% would reduce excretion by only 33% (Table 3).

Finally, we evaluated the sensitivity of these results to the assumption that ciprofloxacin had no effect on resistant bacteria and similar simulations were conduced assuming that the ratio C_50R_/C_50S_ was equal to 4, 16, 100 (see methods). The results did not change substantially when varying the ratio C_50R_/C_50S_ (Table S1). For instance, assuming no effect of ciprofloxacin on resistant bacteria, and a dosing regimen of 15 mg/kg/day for 5 days, we found in the main analysis that drug concentrations would need to be reduced by 90% (i.e., divided by 10) in order to reduce by 50% the amount of excreted resistance. Assuming C_50R_/C_50S_ equal to 4, 16 and 100, the drug concentrations would need to be reduced by ∼75%, 80% and ∼85%, respectively, i.e., the same order of magnitude than in the main analysis.

## Discussion

To our knowledge, the bacterial kinetic model we proposed here is the first one developed on *in vivo* within-host data to characterize the relationship between antibiotic concentrations and resistance to fluoroquinolones in feces. This approach brings new insights on fundamental and clinical aspects of drug resistance.

First, we showed that the proposed mathematical model of resistant and susceptible enterobacteria could well describe the kinetics of both populations during and after treatment. Initiation of treatment led to the rapid elimination of susceptible enterobacteria, with a decline of about 3 log_10_CFU/g in the first 24 h of treatment observed in fecal counts, consistent with the kinetics of *Salmonella* in pigs treated with enrofloxacin [41]. Using a model to fit these data allowed us to estimate the half-life of sensitive enterobacteria during treatment to about 37 minutes. Interestingly, an even more rapid half-life of about 5 minutes was found in *Escherichia coli* exposed to ciprofloxacin [19] but this estimate was found *in vitro* where the metabolism of the bacteria may be different.

The rapid elimination of susceptible enterobacteria created a vast replication space that enabled the proliferation of resistant enterobacteria that remained at high levels in feces for more than 3 weeks after treatment end in both dosing groups, consistent with previous reports [14]. Though an antibiotic effect on resistant enterobacteria cannot be ruled out, our results suggest that treatment has only minimal activity on resistant enterobacteria (i.e., C_50R_≫C_50S_). Because this effect could not be precisely estimated we tested different putative values for the ratio C_50R_/C_50S_ and we found that the best description of the data was obtained when assuming no effect of ciprofloxacin on resistant enterobacteria (i.e., C_50R_/C_50S_ = ∞). Interestingly resistant bacteria were pre-existing to treatment in all piglets. The presence of resistant bacteria can result from *de novo* spontaneous mutations or from continuous ingestion of ciprofloxacin-resistant bacteria. Although the presence of resistance due to de novo mutations in the GT was unlikely (see Methods), both hypotheses were tested, and a better fit was obtained when a continuous ingestion of susceptible and resistant bacteria was assumed rather than mutations (see Supporting Information).

Next, we performed Monte-Carlo simulations in order to estimate the relationships between treatment duration, antibiotic colonic exposure and excretion of resistant enterobacteria. Interestingly we showed that even a one-day exposure to antibiotics, a practice recommended to treat some types of infections [42] or in antibioprophylaxis [43], could induce a 20-fold increase in resistance excretion compared to an untreated piglet. Not surprisingly, for a given level of drug exposure, longer treatment led to higher median total amounts of resistant enterobacteria excreted over an average piglet's lifetime, noted AR. However, this relationship was highly nonlinear. Indeed, for a colonic exposure such as that resulting from a treatment of 15 mg/kg/day (i.e., within the range of a therapeutic dose), AR was equal to 7.5, 8.6 and 9.2 log_10_CFU for 1, 3 and 5 days of exposure, respectively, as compared to 6.2 log_10_CFU in an untreated piglet. The fact that even a low dose of antibiotic can lead to high and sustained levels of resistance for long period of time had already been observed in vitro [44] and confirmed recently in the human feces in vivo of healthy volunteers receiving ciprofloxacin [14]. Thus this approach is highly relevant to predict the effect of reduced length of treatments on antimicrobial resistance and confirms that unnecessary use of antibiotics, even for short period of time, may lead to massive resistance excretion [45], [46]. Therefore by all means the best way to reduce antibiotic selective pressure on intestinal bacteria is to avoid all unnecessary use of antibiotics.

Beside limiting the use of antibiotics, one can also play on the intestinal antibiotic concentration to reduce resistance excretion. In order to achieve about 50% reduction in the quantities of resistant bacteria excreted in the environment, we found that colonic concentrations had to be reduced by 67%, 80% and 90% for 1, 3 and 5 days of treatment, respectively. Importantly, these results did not change substantially when we assumed that ciprofloxacin had also an effect on resistant enterobacteria or when we assumed that resistant enterobacteria were due to mutations (see Supporting Information).

Recently, it has been shown that a charcoal-based specifically chosen adsorbent, formulated to target late ileum and colon to avoid upper tract adsorption of orally administered drugs, could decrease fecal levofloxacin (a FQ) concentrations administered by infusion in dogs, to the extent of 85% [47]. Therefore our prediction that concentrations have to be reduced from 67% to 90% does seem a realistic objective and our results could be highly useful in order to anticipate the impact of these new strategies [17], [18], [48].

The two major limitations of this study were the lack of frequent measurements and the uncertainty associated with these measurements. The use of a sophisticated statistical approach based on non-linear mixed effect models made it possible to precisely estimate most of parameters in spite of a high residual error. More detailed data will be needed to estimate some important parameters such as the proportion of ingested resistant bacteria g_r_/g_s_. Likewise the lack of frequent fecal drug concentrations did not allow for a more physiological PK model. However, the model used here was sufficient to describe the most important feature of ciprofloxacin fecal pharmacokinetics, i.e., the increase in ciprofloxacin concentrations (in the first two 2 days) followed by a plateau. Importantly a similar feature was also reported with enrofloxacin (another fluoroquinolone) in a study where PK was collected twice daily in pigs treated once daily during 5 days [49]. Another important limitation of this study is that our simulation extrapolates to a context of treatment longer than 5 days for which no data was available. Overall future experiments with richer data on larger populations, with different FQ compounds and various dosage regimens will be useful to overcome these limitations and to address other interesting aspects. In particular these studies should include isolation of piglets, control of ingested food and frequent cleaning of the life-place in order to better characterize the origin of resistant enterobacteria and to confirm our result that resistant bacteria in piglets are coming from the environment rather than via direct mutation in the gastrointestinal tract. Lastly, it is yet unknown to what extent these results can be extrapolated to other animal species or to humans. Here, the rapid growth of resistant strains was largely due to the fact that almost all piglets (25/29) had detectable levels of preexisting resistance. This is probably not the case in humans [14] and therefore understanding the growth of resistance in humans will require expanding the model to account for stochastic events such as the spontaneous apparition of resistant enterobacteria [21].

In summary, we proposed here the first approach to model *in vivo* the kinetics of resistant enterobacteria in feces during antibiotic treatment. This approach could be particularly relevant to design and evaluate novel strategies that aim to reduce intestinal exposure to antibiotic residues in order to reduce resistance excretion and dissemination in the environment.

## Supporting Information

Figure S1 Plots of observed values versus individual predicted values by the final model for fecal ciprofloxacin concentrations (left), resistant Enterobacteriaceae counts (center), total Enterobacteriaceae counts (right). Circle symbol corresponds to observed data and cross symbol corresponds to data below the limit of detection (0.1 µg/g for ciprofloxacin concentrations and 2 log_10_CFU/g for enterobacteria counts).
(TIF)

Figure S2 Plots of normalized prediction distribution errors versus the predictions by the final model (left) and versus time (right) for: A) fecal ciprofloxacin concentrations (µg/g), B) resistant enterobacteria counts (log_10_CFU/g), C) total enterobacteria counts (log_10_CFU/g). Blue symbol corresponds to observed data and red symbol corresponds to data below the limit of detection (0.1 µg/g for ciprofloxacin concentrations and 2 log_10_CFU/g for enterobacteria counts).
(TIF)

Figure S3 Resistant (first column), total (second column) and susceptible (third column) enterobacteria predicted from Day 1 to Day 60 for various fecal concentrations of ciprofloxacin *C_ss_*: 0 µg/g (black), 0.9 µg/g (grey), 1.8 µg/g (violet), 2.9 µg/g (blue), 8.7 µg/g (green), 87 µg/g (red) for different treatment durations: A) 1 day, B) 3 days, C) 5 days; D) 10 days.
(TIF)

Table S1 Prediction of impact of ciprofloxacine colonic exposure and treatment duration on the amount of resistant enterobacteria (R) excreted over 150 days with different models.
(DOC)

Text S1 Sensitivity of the initial conditions with respect to model parameters.
(DOC)

Text S2 Code of the final model implemented in MONOLIX software.
(DOC)

Text S3 Alternative models with mutation.
(DOC)
