# Supplementary figures and images for: Mathematical Modeling of Bacterial Kinetics to Predict the Impact of Antibiotic Colonic Exposure and Treatment Duration on the Amount of Resistant Enterobacteria Excreted
Source: PLoS Comput Biol. 2014 Sep 11;10(9):e1003840. doi: 10.1371/journal.pcbi.1003840 (PMC4161292; doi:10.1371/journal.pcbi.1003840)

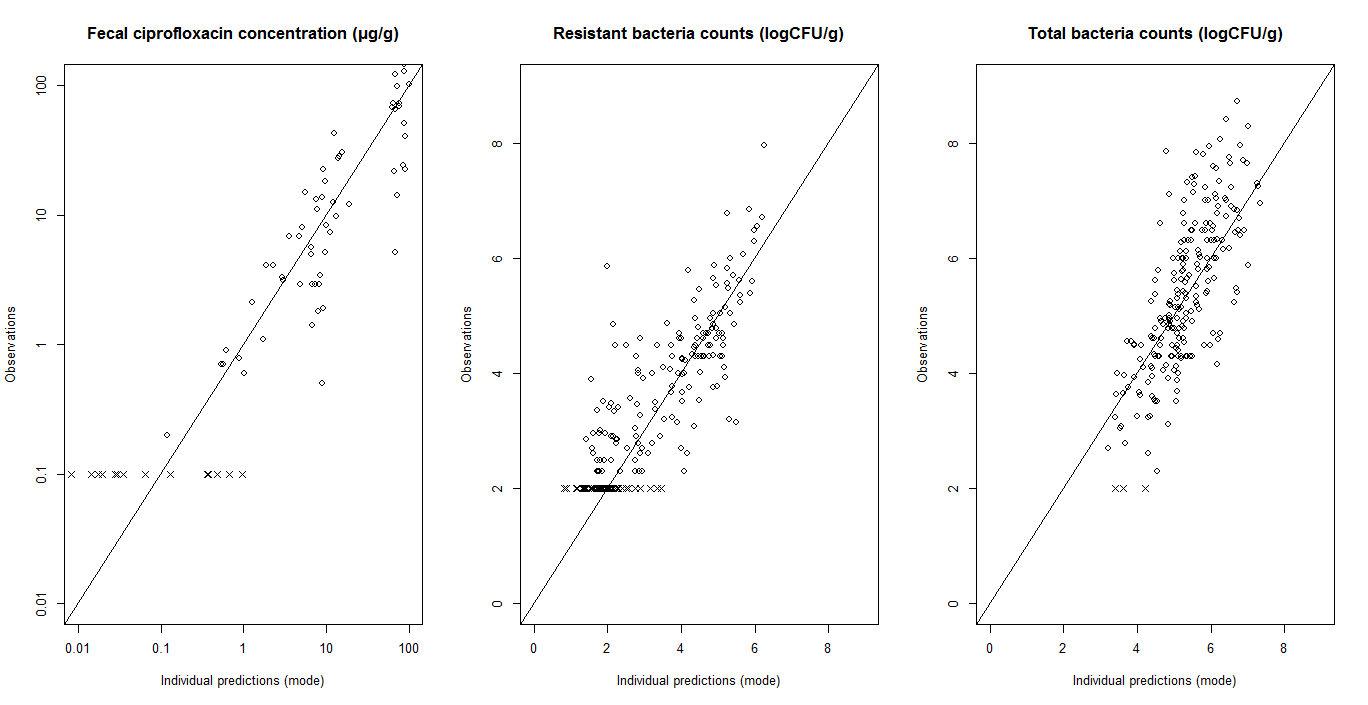

Supplement: Figure S1 — Plots of observed values versus individual predicted values by the final model for fecal ciprofloxacin concentrations (left), resistant Enterobacteriaceae counts (center), total Enterobacteriaceae counts (right). Circle symbol corresponds to observed data and cross symbol corresponds to data below the limit of detection (0.1 µg/g for ciprofloxacin concentrations and 2 log10CFU/g for enterobacteria counts). (TIF) [file pcbi.1003840.s001.tif]

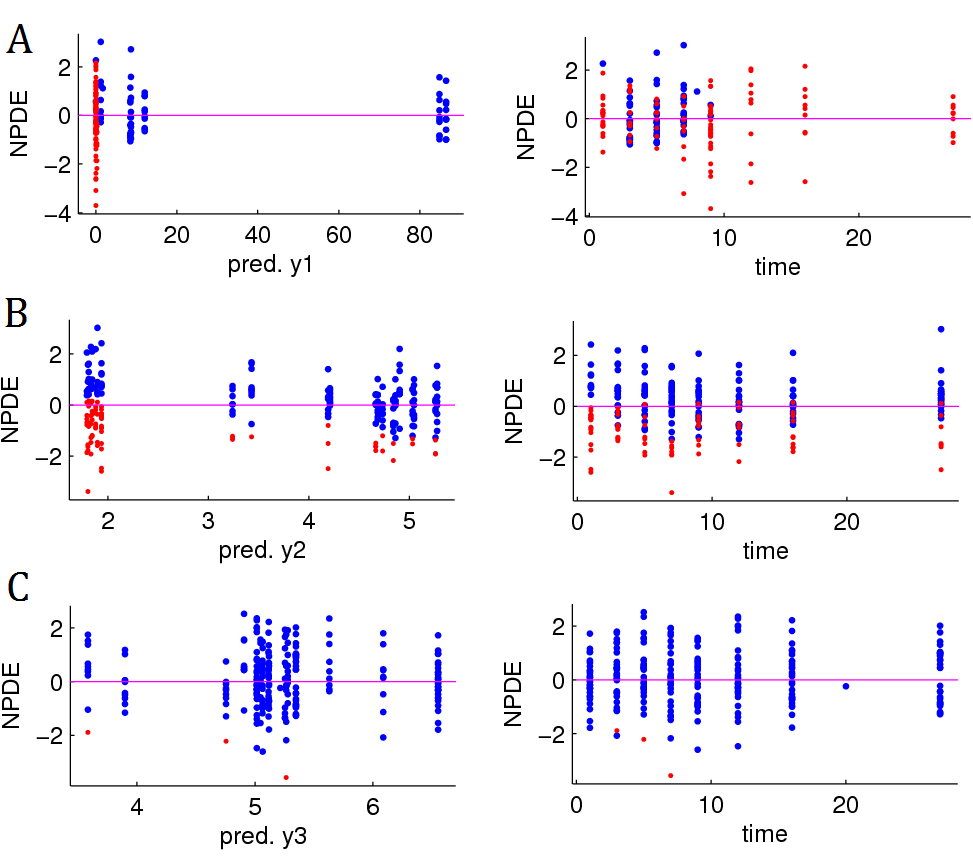

Supplement: Figure S2 — Plots of normalized prediction distribution errors versus the predictions by the final model (left) and versus time (right) for: A) fecal ciprofloxacin concentrations (µg/g), B) resistant enterobacteria counts (log10CFU/g), C) total enterobacteria counts (log10CFU/g). Blue symbol corresponds to observed data and red symbol corresponds to data below the limit of detection (0.1 µg/g for ciprofloxacin concentrations and 2 log10CFU/g for enterobacteria counts). (TIF) [file pcbi.1003840.s002.tif]

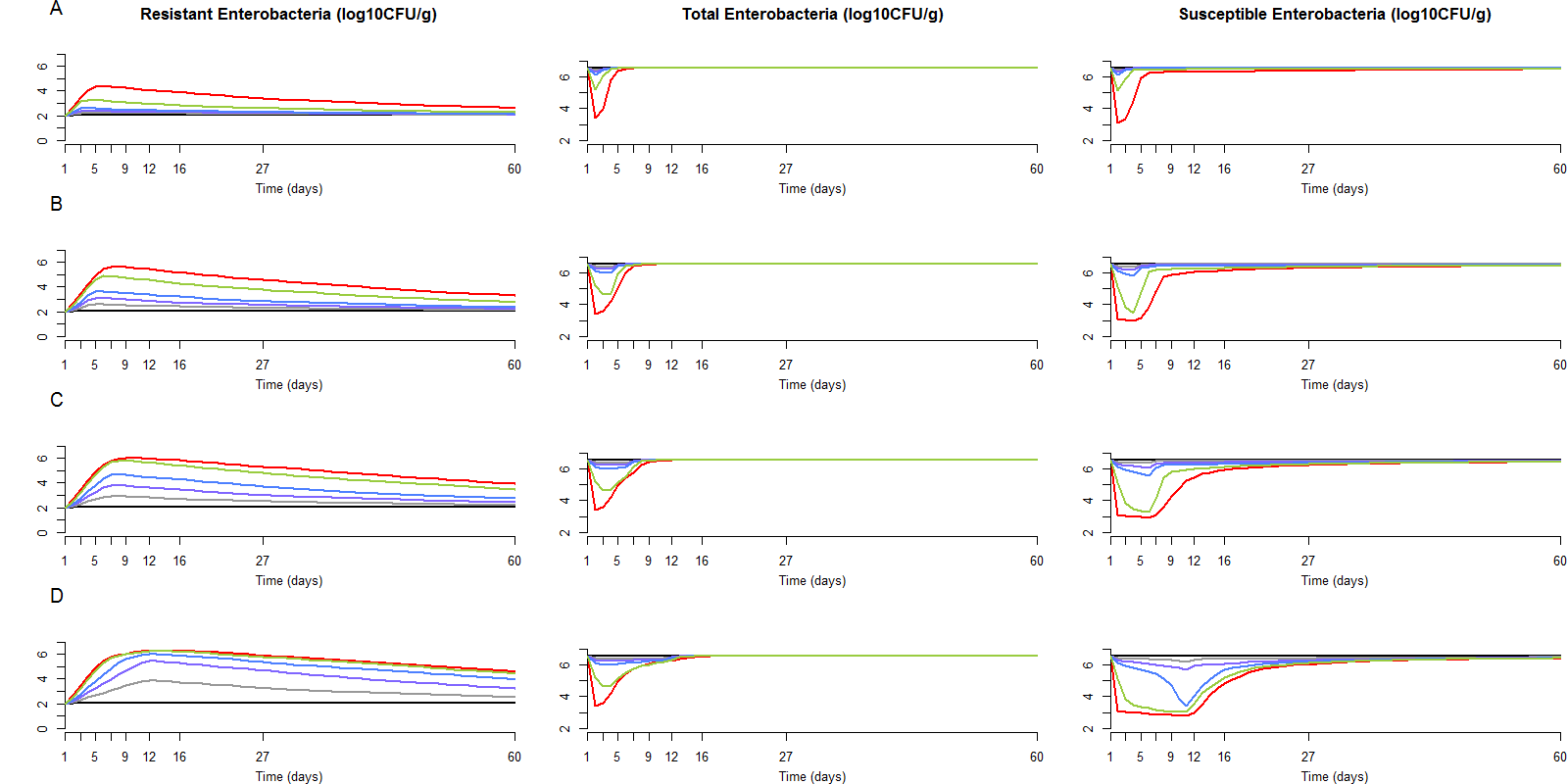

Supplement: Figure S3 — Resistant (first column), total (second column) and susceptible (third column) enterobacteria predicted from Day 1 to Day 60 for various fecal concentrations of ciprofloxacin Css: 0 µg/g (black), 0.9 µg/g (grey), 1.8 µg/g (violet), 2.9 µg/g (blue), 8.7 µg/g (green), 87 µg/g (red) for different treatment durations: A) 1 day, B) 3 days, C) 5 days; D) 10 days. (TIF) [file pcbi.1003840.s003.tif]
