# Supplementary material for: Mathematical Modeling of Bacterial Kinetics to Predict the Impact of Antibiotic Colonic Exposure and Treatment Duration on the Amount of Resistant Enterobacteria Excreted
Source: PLoS Comput Biol. 2014 Sep 11;10(9):e1003840. doi: 10.1371/journal.pcbi.1003840 (PMC4161292; doi:10.1371/journal.pcbi.1003840)
Supplement: Table S1 — Prediction of impact of ciprofloxacine colonic exposure and treatment duration on the amount of resistant enterobacteria (R) excreted over 150 days with different models. (DOC) [file pcbi.1003840.s004.doc]

**Table S1: Prediction of impact of ciprofloxacine colonic exposure and treatment duration on the amount of resistant enterobacteria (R) excreted over 150 days with different models**

| Treatment  duration | C_ss_  (µg/g) | % of C_ss_  reduction * | Total amount of R excreted in logCFU  (% of reduction *) | | | | |
| --- | --- | --- | --- | --- | --- | --- | --- |
|  |  |  | C_50R_ /C_50S_ = 4 | C_50R_ /C_50S_ =16 | C_50R_ /C_50S_ =100 | **no effect against R** | with mutation (MR2) |
| 1 day | 87 | 0 | 9.0 (0) | 9.2 (0) | 8.5 (0) | **7.5 (0)** | 7.7 (0) |
|  | 58 | 33 | 8.3 (81.8) | 8.5 (81.3) | 8.3 (42.2) | **7.4 (19.7)** | 7.5 (25.1) |
|  | 29 | 67 | 7.2 (98.5) | 7.1 (99.4) | 7.6 (89.2) | **7.2 (46.8)** | 7.3 (58.7) |
|  | 18 | 80 | 6.9 (99.6) | 6.5 (99.9) | 7.0 (97.6) | **7.1 (61.0)** | 7.0 (77.3) |
|  | 8.7 | 90 | 6.6 (99.9) | 6.3 (100) | 6.6 (99.6) | **6.6 (90.4)** | 6.6 (91.6) |
|  | 2.9 | 97 | 6.5 (100) | 6.2 (100) | 6.4 (99.9) | **6.3 (98.1)** | 6.0 (98.0) |
|  | 1.8 | 98 | 6.5 (100) | 6.2 (100) | 6.4 (99.9) | **6.3 (99.0)** | 5.8 (98.9) |
|  | 0.9 | 99 | 6.5 (100) | 6.2 (100) | 6.3 (100) | **6.3 (99.6)** | 5.6 (99.5) |
|  | 0 | 100 | 6.5 (100) | 6.2 (100) | 6.3 (100) | **6.2 (100)** | 5.2 (100) |
| 3 days | 87 | 0 | 10.1 (0) | 9.9 (0) | 9.5 (0) | **8.6 (0)** | 8.8 (0) |
|  | 58 | 33 | 10.0 (11.9) | 9.9 (6.9) | 9.5 (0) | **8.6 (4.8)** | 8.7 (23.0) |
|  | 29 | 67 | 9.4 (78.7) | 9.6 (57.0) | 9.4 (25.6) | **8.4 (42.3)** | 8.5 (52.3) |
|  | 18 | 80 | 8.3 (98.3) | 8.6 (95.4) | 9.0 (68.6) | **8.3 (50.4)** | 8.3 (68.2) |
|  | 8.7 | 90 | 7.2 (99.9) | 7.0 (99.9) | 7.8 (98.2) | **7.8 (84.4)** | 8.0 (85.9) |
|  | 2.9 | 97 | 6.7 (100) | 6.3 (100) | 6.6 (99.6) | **6.9 (98.5)** | 7.0 (98.6) |
|  | 1.8 | 98 | 6.6 (100) | 6.3 (100) | 6.4 (100) | **6.5 (99.5)** | 6.5 (99.5) |
|  | 0.9 | 99 | 6.5 (100) | 6.2 (100) | 6.4 (100) | **6.3 (99.9)** | 6.1 (99.9) |
|  | 0 | 100 | 6.5 (100) | 6.2 (100) | 6.3 (100) | **6.2 (100)** | 5.2 (100) |
| 5 days | 87 | 0 | 10.1 (0) | 10.0 (0) | 9.8 (0) | **9.2 (0)** | 9.5 (0) |
|  | 58 | 33 | 10.1 (0) | 10.0 (0) | 9.8 (0) | **9.2 (3.5)** | 9.5 (7.3) |
|  | 29 | 67 | 10.0 (23.2) | 9.9 (14.0) | 9.8 (0) | **9.1 (17.7)** | 9.4 (20.7) |
|  | 18 | 80 | 9.5 (75.9) | 9.6 (52.8) | 9.7 (11.9) | **9.0 (33.0)** | 9.3 (32.7) |
|  | 8.7 | 90 | 8.0 (99.3) | 8.2 (98.5) | 9.1 (79.3) | **8.8 (57.5)** | 9.1 (57.8) |
|  | 2.9 | 97 | 6.8 (100) | 6.4 (100) | 6.9 (99.9) | **7.8 (96.3)** | 8.0 (97.1) |
|  | 1.8 | 98 | 6.7 (100) | 6.3 (100) | 6.6 (100) | **7.0 (99.4)** | 7.2 (99.5) |
|  | 0.9 | 99 | 6.6 (100) | 6.3 (100) | 6.4 (100) | **6.5 (99.9)** | 6.4 (99.9) |
|  | 0 | 100 | 6.5 (100) | 6.2 (100) | 6.3 (100) | **6.2 (100)** | 5.2 (100) |
| 10 days | 87 | 0 | 10.2 (0) | 10.1 (0) | 9.9 (0) | **9.6 (0)** | 9.8 (0) |
|  | 58 | 33 | 10.2 (0) | 10.1 (0) | 9.9 (0) | **9.6 (2.5)** | 9.8 (1.8) |
|  | 29 | 67 | 10.2 (0) | 10.1 (0) | 9.9 (0) | **9.5 (4.6)** | 9.8 (4.8) |
|  | 18 | 80 | 10.1 (7.9) | 10.0 (0) | 9.9 (0) | **9.5 (16.9)** | 9.8 (7.2) |
|  | 8.7 | 90 | 9.6 (73.3) | 9.7 (8) | 9.8 (17.5) | **9.5 (17.0)** | 9.8 (12.2) |
|  | 2.9 | 97 | 7.3 (99.9) | 7.1 (99.9) | 8.2 (98.2) | **9.1 (63.0)** | 9.5 (50.6) |
|  | 1.8 | 98 | 6.9 (100) | 6.5 (100) | 7.1 (99.9) | **8.5 (91.5)** | 8.9 (89.0) |
|  | 0.9 | 99 | 6.6 (100) | 6.3 (100) | 6.6 (100) | **7.1 (99.7)** | 7.4 (99.6) |
|  | 0 | 100 | 6.5 (100) | 6.2 (100) | 6.3 (100) | **6.2 (100)** | 5.2 (100) |

C_ss_: fecal plateau concentration of ciprofloxacin

*: compared to the value achieved with a dose of 15 mg/kg/day
