# Supplementary material for: Mathematical Modeling of Bacterial Kinetics to Predict the Impact of Antibiotic Colonic Exposure and Treatment Duration on the Amount of Resistant Enterobacteria Excreted
Source: PLoS Comput Biol. 2014 Sep 11;10(9):e1003840. doi: 10.1371/journal.pcbi.1003840 (PMC4161292; doi:10.1371/journal.pcbi.1003840)
Supplement: Text S1 — Sensitivity of the initial conditions with respect to model parameters. (DOC) [file pcbi.1003840.s005.doc]

**Text S1.** **Sensitivity of the initial conditions with respect to model parameters**

At baseline, the counts per gram of feces of ciprofloxacin resistant enterobacteria, R(t), and drug susceptible enterobacteria, S(t) are equal to S_0_ and R_0_, respectively:

$$\begin{matrix} S_{0}=N_{0}\frac{(\alpha_{S}-k_{T})+\sqrt{\Delta}}{2\alpha_{S}}\text{ with }\Delta=(k_{T}-\alpha_{S})^{2}+4\frac{\alpha_{S}g_{S}}{N_{0}} \\ R_{0}=\frac{g_{R}}{k_{T}-\alpha_{R}\left(1-\frac{S_{0}}{N_{0}}\right)} \end{matrix}$$

We can approximate S_0_ and R_0_ as

$$\begin{matrix} S_{0}\approx N_{0}\frac{(\alpha_{S}-k_{T})+\sqrt{(k_{T}-\alpha_{S})^{2}}}{2\alpha_{S}}\text{ }\approx\frac{2(\alpha_{S}-k_{T})}{2\alpha_{S}}N_{0}\approx N_{0} because \alpha_{S}>>k_{T} \\ R_{0}\approx\frac{g_{R}}{k_{T}} because S_{0}\approx N_{0} \end{matrix}$$
