# Supplementary material for: Mathematical Modeling of Bacterial Kinetics to Predict the Impact of Antibiotic Colonic Exposure and Treatment Duration on the Amount of Resistant Enterobacteria Excreted
Source: PLoS Comput Biol. 2014 Sep 11;10(9):e1003840. doi: 10.1371/journal.pcbi.1003840 (PMC4161292; doi:10.1371/journal.pcbi.1003840)
Supplement: Text S2 — Code of the final model implemented in MONOLIX software. (DOC) [file pcbi.1003840.s006.doc]

**Text S2. Code of the final model implemented in MONOLIX software**

DESCRIPTION: Ciprofloxacin PK/PD model (resistant & total Enterobacteria)

INPUT:

parameter = {V, k, lN0, a, b, alpha_s, alpha_r, g_s, g_r, k_t, delta_m, C50s, h}

EQUATION:

C = pkmodel(V,k)

t0=1

N=10^(lN0+log10(exp(-a*(t-1))-exp(-b*(t-1))+1))

delta=(k_t-alpha_s)^2+4*alpha_s*g_s/(10^lN0)

S0=(10^lN0)*(alpha_s-k_t+sqrt(delta))/(2*alpha_s)

R0=g_r/(k_t-alpha_r*(1-S0/(10^lN0)))

R_0=R0

S_0=S0

ddt_S = alpha_s*S*(1-(R+S)/N) + g_s - k_t*S - (delta_m*C^h/(C^h+C50s^h))*S

ddt_R = alpha_r*R*(1-(R+S)/N) + g_r - k_t*R

Conc = C

logS=log10(S)

logR=log10(R)

logtot=log10(R+S)

OUTPUT:

output = {Conc, logR, logtot}
