# Supplementary material for: Mathematical Modeling of Bacterial Kinetics to Predict the Impact of Antibiotic Colonic Exposure and Treatment Duration on the Amount of Resistant Enterobacteria Excreted
Source: PLoS Comput Biol. 2014 Sep 11;10(9):e1003840. doi: 10.1371/journal.pcbi.1003840 (PMC4161292; doi:10.1371/journal.pcbi.1003840)
Supplement: Text S3 — Alternative models with mutation. (DOC) [file pcbi.1003840.s007.doc]

**Text S3. Alternative models with mutation**

We have tested two alternative models assuming that the source of resistant bacteria was mutation from S to R with rate *µ* rather than continuous incoming, i.e g_r_ = 0:

**Model MR1 assumes constant rate *µ* from S to R**

$$\begin{matrix} \frac{dS}{dt}=\alpha_{S}(1-\mu)\left(1-\frac{S+R}{N_{\max}}\right)S+g_{S}-k_{T}S-\delta_{\max}\frac{C}{C+C_{50}}S \\ \frac{dR}{dt}=\alpha_{R}\left(1-\frac{S+R}{N_{\max}}\right)R-k_{T}R+\alpha_{S}\mu\left(1-\frac{S+R}{N_{\max}}\right)S \\ N_{\max}(t)=N_{0}\left(e^{-at}-e^{-bt}+1\right) \end{matrix}$$

Initial conditions (C_0_ = 0 and R_0_ « S_0_)

$$\begin{matrix} S_{0}=N_{0}\frac{(\alpha_{S}(1-\mu)-k_{T})+\sqrt{\Delta}}{2\alpha_{S}}\text{ with }\Delta=(k_{T}-\alpha_{S}(1-\mu))^{2}+4\frac{\alpha_{S}(1-\mu)g_{S}}{N_{0}} \\ R_{0}=\frac{\alpha_{S}\mu\left(\frac{1-S_{0}}{N_{0}}\right)S_{0}}{k_{T}-\alpha_{R}\left(\frac{1-S_{0}}{N_{0}}\right)} \end{matrix}$$

**Model MR2 assumes a ciprofloxacin-dependent rate from S to R**

$$\begin{matrix} \frac{dS}{dt}=\alpha_{S}\left(1-\frac{S+R}{N_{\max}}\right)S+g_{S}-k_{T}S-\delta_{\max}\frac{C}{C+C_{50}}S-\mu\left(\alpha_{S}\left(1-\frac{S+R}{N_{\max}}\right)+\lambda\frac{C}{C+C_{50}}\right)S \\ \frac{dR}{dt}=\alpha_{R}\left(1-\frac{S+R}{N_{\max}}\right)R-k_{T}R+\mu\left(\alpha_{S}\left(1-\frac{S+R}{N_{\max}}\right)+\lambda\frac{C}{C+C_{50}}\right)S \\ N_{\max}(t)=N_{0}\left(e^{-at}-e^{-bt}+1\right) \end{matrix}$$

Initial conditions (C_0_ = 0 and R_0_ « S_0_)

$$\begin{matrix} S_{0}=N_{0}\frac{(\alpha_{S}(1-\mu)-k_{T})+\sqrt{\Delta}}{2\alpha_{S}}\text{ with }\Delta=(k_{T}-\alpha_{S}(1-\mu))^{2}+4\frac{\alpha_{S}(1-\mu)g_{S}}{N_{0}} \\ R_{0}=\frac{\alpha_{S}\mu\left(\frac{1-S_{0}}{N_{0}}\right)S_{0}}{k_{T}-\alpha_{R}\left(\frac{1-S_{0}}{N_{0}}\right)} \end{matrix}$$

We fitted these two models to the experimental data and found the following values:

|  | MR1 | MR2 |
| --- | --- | --- |
| *µ* | 3.26 10^-9^ | 6.13 10^-10^ |
| λ | NA | 1.03 |
| BIC | 1978.12 | 1977.91 |

None of these models improved data fitting compared to our final model that neglected the effect of mutations (BIC = 1954).

Simulations were also carried out with the estimated parameter values found with model MR2. The results did not change substantially compared to the final model (Table S1).
